# Predictive Factors of Potential Malignant Transformation in Recurrent Calcifying Cystic Odontogenic Tumor: Review of the Literature

**DOI:** 10.1155/2013/853095

**Published:** 2013-07-15

**Authors:** Sepideh Mokhtari, Zhaleh Mohsenifar, Maedeh Ghorbanpour

**Affiliations:** ^1^Department of Oral and Maxillofacial Pathology, School of Dentistry, Shahid Beheshti University of Medical Sciences, Tehran 1983969411, Iran; ^2^Department of Pathology, Taleghani Hospital, Shahid Beheshti University of Medical Sciences, Tehran 1983969411, Iran

## Abstract

Calcifying cystic odontogenic tumor (CCOT) demonstrates considerable diversity in histopathology and clinical behavior. Ghost cell odontogenic carcinoma (GCOC) is the rare malignant counterpart of CCOT and it frequently arises from malignant transformation of a recurrent CCOT. In this paper, we present a case of CCOT and discuss its distinct histopathologic features in recurrence. Then, we will have a review on clinical, histopathological, and immunohistochemical aspects of GCOC in the literature. Predictive factors of malignant transformation in a benign CCOT will also be discussed.

## 1. Introduction

Calcifying odontogenic cyst (COC) represents 2% of all odontogenic lesions in the jaw [1]. It demonstrates considerable diversity in histopathology and clinical behavior. Latest subclassification of World Health Organization (WHO) has renamed this lesion as calcifying cystic odontogenic tumor (CCOT) [2]. Ghost cell odontogenic carcinoma (GCOC) is the malignant counterpart of this tumor and it frequently arises from malignant transformation of CCOT after multiple recurrences [3]. Here, we present a case of recurrent CCOT and discuss its distinct histopathologic features as potential predictive factors of future malignancy. We will also have a review on clinical, histopathological, and immunohistochemical characteristics of GCOC in the literature. 

## 2. Case Presentation

**Figure 1:**
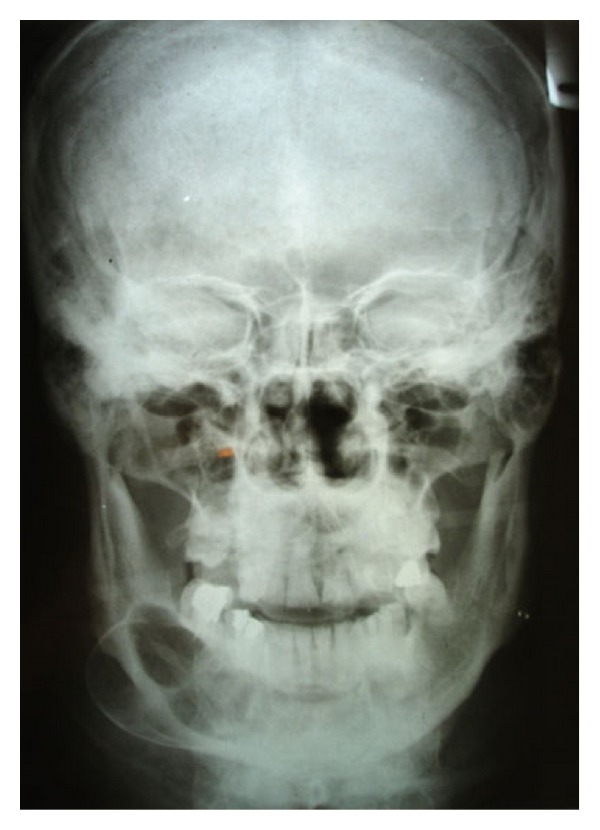
Posterior-anterior view of primary tumor shows a multilocular radiolucent lesion.

**Figure 2:**
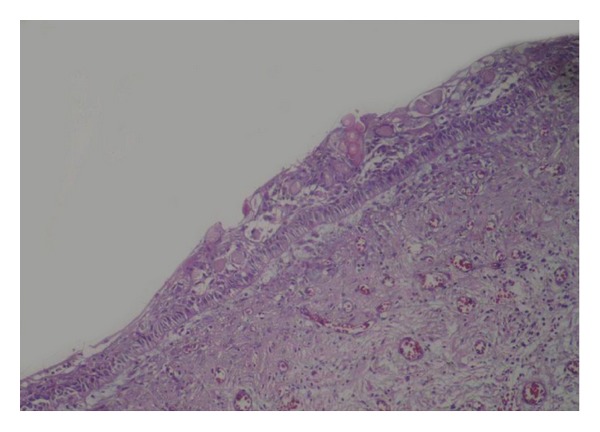
Photomicrograph of the cystic lesion lined by odontogenic epithelium (resembling ameloblasts), stellate reticulum, and ghost cells (H&E).

**Figure 3:**
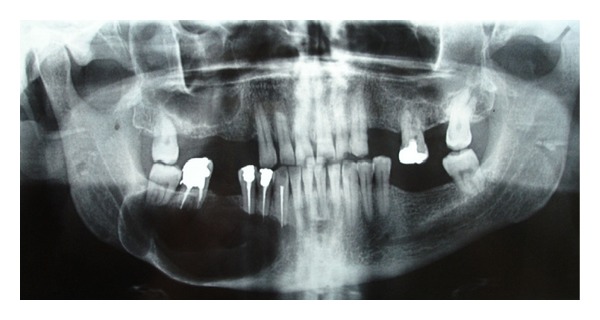
Panoramic radiograph; 2 weeks after operation.

**Figure 4:**
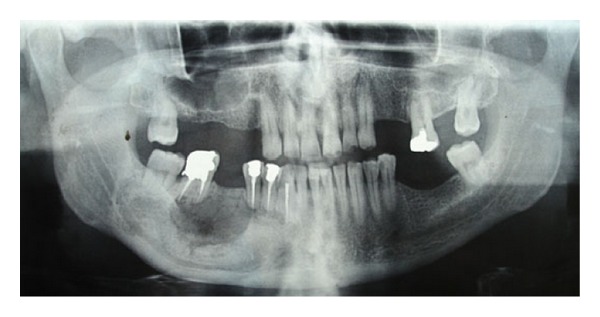
Panoramic radiograph; 18 months after operation.

**Figure 5:**
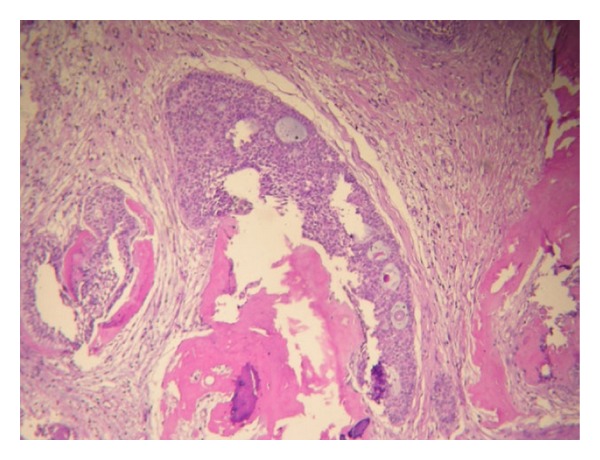
Photomicrograph of the recurrent lesion with tumoral cribriform proliferations and dentinoid material in the cyst wall (H&E).

**Figure 6:**
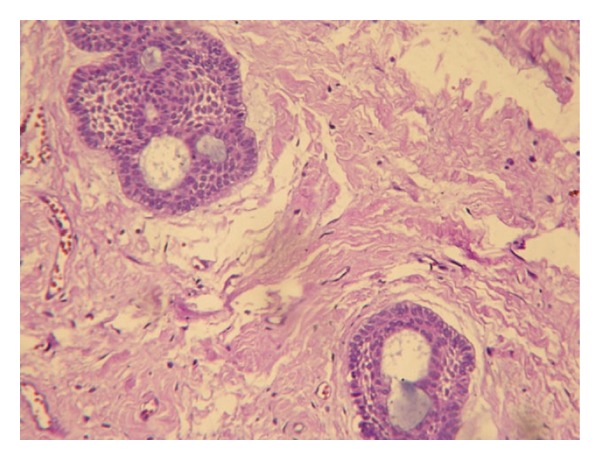
Photomicrograph of the recurrent lesion with cribriform proliferations (H&E).

**Figure 7:**
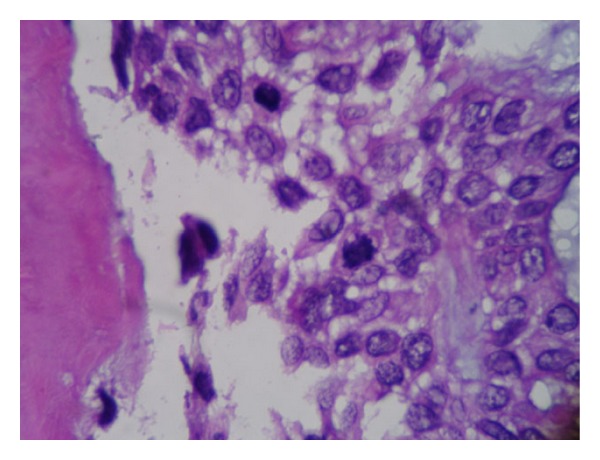
Photomicrograph of the recurrent lesion shows mitotic figures (H&E).

A 54-year-old male presented with swelling in the right side of mandible. He had a history of right first molar extraction 5 years ago with subsequent abscess formation and without any treatment. Radiographic examination revealed a multilocular radiolucent lesion (Figure 1). Root resorption of right mandibular canine and premolars was also obvious. Incisional biopsy revealed a benign cystic lesion with typical histologic features of calcifying odontogenic cyst (Figure 2). The lesion was excised and extensively curetted. Serial panoramic radiographs were taken in 2-week, 3-, 11-, 13-, and 18-month follow-up (Figures 3 and 4). Continuous healing process was seen in panoramic views. However, in all radiographs a nonhealing radiolucent area with progressive increase in size was evident. This area was apparent in the radiograph of 18-month follow-up as a well-defined radiolucent lesion. Clinical examination revealed a swelling in the right side of mandible measuring 4 × 3 cm. The surface skin was intact with no erythema or tenderness and the patient had no lymphadenopathy. In computed tomographic sections, buccal and lingual cortex perforations were evident. Tumor recurrence was confirmed by histopathologic evaluation. However, in contrast to the initial lesion, the recurrent cystic lesion had tumoral proliferations in the cyst wall (Figure 5). Tumoral nests contained ghost cells and dentinoid material, some representing a cribriform pattern (Figure 6). Scattered mitotic figures and mild atypia were observed (Figure 7). Granulomatous reaction and foreign body type giant cells were also present throughout the lesion.

**Figure 8:**
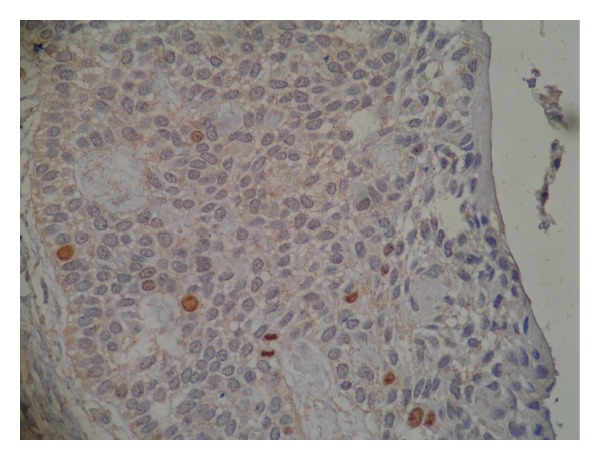
Immunohistochemical staining for Ki-67 in the recurrent case. One mitotic figure in anaphase stage with intense staining is also present.

**Figure 9:**
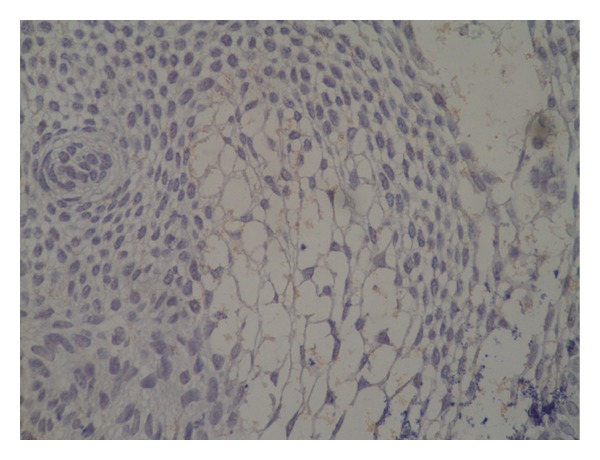
Immunohistochemical staining for p53 in the recurrent case. Very few cells are positive for p53.

The primary and recurrent cases went through microscopic evaluation with immunohistochemistry including p53 and Ki-67. P53 staining was negative in both cases whereas Ki-67 labeling index was increased in the recurrent case with a mean of 5% in cribriform epithelial nests, confirming the proliferative activity of recurrent case (Figures 8 and 9). Therefore, the second lesion was diagnosed as benign recurrent CCOT with histopathologic and immunohistochemical evidence of aggressive behavior.

## 3. Discussion

GCOC is the rare malignant counterpart of CCOT and approximately, 30 cases have been reported in the literature. GCOC is diagnosed on the basis of atypical histologic features, necrosis, prominent mitoses, infiltrative growth pattern, aggressive behavior, and high expression of Ki-67 and p53 [4]. This malignant odontogenic tumor arises de novo or secondary to its benign counterpart [5]. As previous studies show, the most probable mechanism of GCOC development is malignant transformation in CCOT after multiple recurrences [5, 6]. 

CCOT has no distinct predilection to maxilla or mandible and is slightly more common in women [7]. In contrast, recurrent CCOT [8] and GCOC are more common in maxilla and male patients [5]. In fact, obtaining an adequate surgical margin is difficult in maxilla. Therefore, the recurrence rate and risk of subsequent malignant transformation are increased in maxillary tumors. In addition, odontogenic myxomas, calcifying epithelial odontogenic tumor and ameloblastoma of the posterior maxilla, are particularly dangerous lesions and behave in a more aggressive manner than mandibular cases [7]. 

Table 1 provides a concise review of the literature on clinical and pathological characteristics of GCOC [3, 5, 6, 9–17]. Here, the recurrent case was considered a benign lesion. However, some distinct histopathologic features were present. The primary lesion and its recurrence were both cystic. Nevertheless, recurrent COC had tumoral proliferations in the form of cribriform nests in the cyst wall. Mild pleomorphism and hyperchromatism were also present and mitotic activity was increased. Some authors have observed these features in recurrent cases of CCOT with subsequent malignant transformation [4, 6, 18]. Li and Gao presented a case of CCOT in the maxilla, which transformed to ghost cell odontogenic carcinoma after multiple recurrences. After each recurrence, cribriform structures were larger; mitotic rate was higher and pleomorphism was more evident [6]. In addition, Motosugi et al. reported a case of malignant transformation in recurrent CCOT. They observed elevated Ki-67 and p53 expression in the recurrent lesions [4]. Li et al. reported a case of CCOT, which transformed into GCOC in first recurrence. They noticed proliferative cribriform nests in tumor histopathology and infiltrative aggressive behavior clinically [12]. Therefore, it seems that some recurrent cases of CCOT have distinct histopathologic features that could be noticed as predictive factors for aggressive behavior or malignant transformation in the future. 

We observed increased Ki-67 labeling index in cribriform nests of recurrent case. However, p53 expression was rarely seen in tumor cells. Some authors have performed immunohistochemical examinations in CCOT and GCOC as its malignant counterpart.  Table 2 presents a review of these literatures [4, 12, 13, 19–25]. Many investigators have evaluated the expression of Ki-67 as a biomarker of cell proliferation activity in CCOT and GCOC. In almost all literatures ki-67 expression was weak in CCOT but strong in GCOC [4, 12, 19–21]. In addition, MMP-9, as a biomarker of tumor invasion [19–22], and TIMP-1 [22] were more strongly expressed in GCOC than CCOT. In addition, strong expression of p53 in GCOC has been observed in some literatures [4, 24]. Nevertheless, more investigations are required to determine the useful immunohistochemical markers that can be used to find tumors with high recurrence rate and greater possibility of malignant transformation. 

To conclude, malignant transformation should be considered in all recurrent CCOTs particularly in maxillary lesions and male patients [5]. Moreover, CCOTs with cribriform nests, high Ki-67 expression, increased atypia, and mitotic rate are aggressive neoplasms and have a greater probability of malignant transformation in future. Therefore, the pathologists should point out these histopathologic features in pathology reports and its clinical importance to the surgeons. 

CCOT is a benign lesion and most surgeons enucleate the lesion and curette 1-2 mm of the surrounding bone to remove any tumor remnant [26]. However, recurrences are frequently seen in some cases. Therefore, mentioned clinical and histopathological features warrant more radical surgery in some cases. 

Malignant transformation can occur in CCOT rapidly or after a long time. Arashiyama et al. reported a case of calcifying odontogenic cyst that transformed to malignancy after eighteen years [5]. Therefore, long-term careful follow-up of the patients is also recommended.

## Figures and Tables

**Table 1:** Concise review of the literature on clinical and pathological characteristics of GCOC.

Ghost cell odontogenic carcinoma	
Clinical Features	*Tumor Type:* an extremely rare malignant odontogenic tumor
	*Age range:* 13–72 years, mean: 40 years [5, 9]
	*Sex:* male predominance [9, 10].
	*Location:* more common in maxilla than mandible [5, 9, 10].
	*Racial tendency:* Asians [9, 10].
	*Onset:* rapid onset [3, 11] or a long time [5] after excision of CCOT.
	*Sign and Symptoms *
	(i) Painful swelling [3, 12] with local paraesthesia: the most common symptom [13].
	(ii) Some ulcerative with bleeding on contact [14].
	(ii) Sometimes pain is the initial presentation [15].
	(iv) Root resorption (31%) [9].
	(v) Tooth displacements (21%) [9].

Origin	Malignant transformation of a preexisting benign CCOT [5, 10] or other odontogenic tumors [10], Denovo.

Histopathology	*Gross:* cystic [16] or solid.
	(i) Small basaloid cells or large epithelial cells [10].
	(ii) Ghost cells are hard to find and even disappear [6].
	(iii) Frequent presence of benign CCOT separated or admixed with malignant component.

Radiographic appearance	Mixed radiolucent and radiopaque pattern more frequent than radiolucent lesions [9].
	90% with poorly defined borders and 11% well defined [9].

Behavior	16% mortality of local invasiveness [14] or distant metastasis (pulmonary,…) [5, 17].
	Unpredictable course, some indolent and other potentially fatal [10].

Treatment	Radical surgery.

**Table 2:** A review of performed immunohistochemical examinations in CCOT and GCOC in the literature.

	Zhu et al. (2012) [19]	Li et al. (2011) [12]	Gong et al. (2009, 2006) [20, 21]	Motosugi et al. (2009) [4]	Roh et al. (2008) [13]	Geng et al. (2008) [22]	Kim et al. 2000 [23]	Folpe et al. (1998) [24]	Piattelli et al. (1998) [25]
CCOT									
Ki-67	W	W	W	W					S
P53				W					W
CK5 & CK14		P							
CK18		N							
TIMP-1						W			
MMP-9	W in T W in St W in G		W in T W in St			W			
NF-kappaB			S in CT W in NT						
BCL2									S
GCOC									
Ki-67	S	S	S	S					
P53				S				P	
CK5 & CK14		P							
EMA & NSE								P	
CK18		N							
TIMP-1						S			
MMP-9	W in T S in St W in G		W in T S in St			S			
TRAP & VR					N in T P in G				
INVOLUCRN CK, BCLX_L_							P in T N in G		
BCL2							N in T N in G		
BAX							N in T P in G		

MMP: matrix metalloproteinase

TIMP: tissue inhibitor of metalloproteinase

VR: vitronectin receptor

TRAP: tartrate-resistant acid phosphatase

T: tumor

St: stroma

G: ghost cell

P: positive

N: negative

W: weak

S: strong

CT: cytoplasm of tumor cells

NT: nucleus of tumor cells.
